# Income in Adult Survivors of Childhood Cancer

**DOI:** 10.1371/journal.pone.0155546

**Published:** 2016-05-23

**Authors:** Laura Wengenroth, Grit Sommer, Matthias Schindler, Ben D. Spycher, Nicolas X. von der Weid, Eveline Stutz-Grunder, Gisela Michel, Claudia E. Kuehni, Swiss Paediatric Oncology Group (SPOG)

**Affiliations:** 1 Swiss Childhood Cancer Registry, Institute of Social and Preventive Medicine, University of Bern, Bern, Switzerland; 2 Institute of Occupational, Social and Environmental Medicine, Ludwig-Maximilians-Universität, Munich, Germany; 3 Pediatric Hematology/Oncology Unit, University Children’s Hospital Basel (UKBB), University of Basel, Basel, Switzerland; 4 Pediatric Hematology/Oncology Unit, University Hospital Bern (Inselspital), Bern Switzerland; 5 Department of Health Sciences and Health Policy, University of Lucerne, Lucerne, Switzerland; University Hospital Oldenburg, GERMANY

## Abstract

**Introduction:**

Little is known about the impact of childhood cancer on the personal income of survivors. We compared income between survivors and siblings, and determined factors associated with income.

**Methods:**

As part of the Swiss Childhood Cancer Survivor Study (SCCSS), a questionnaire was sent to survivors, aged ≥18 years, registered in the Swiss Childhood Cancer Registry (SCCR), diagnosed at age <21 years, who had survived ≥5 years after diagnosis of the primary tumor. Siblings were used as a comparison group. We asked questions about education, profession and income and retrieved clinical data from the SCCR. We used multivariable logistic regression to identify characteristics associated with income.

**Results:**

We analyzed data from 1’506 survivors and 598 siblings. Survivors were less likely than siblings to have a high monthly income (>4’500 CHF), even after we adjusted for socio-demographic and educational factors (OR = 0.46, p<0.001). Older age, male sex, personal and parental education, and number of working hours were associated with high income. Survivors of leukemia (OR = 0.40, p<0.001), lymphoma (OR = 0.63, p = 0.040), CNS tumors (OR = 0.22, p<0.001), bone tumors (OR = 0.24, p = 0.003) had a lower income than siblings. Survivors who had cranial irradiation, had a lower income than survivors who had no cranial irradiation (OR = 0.48, p = 0.006).

**Discussion:**

Even after adjusting for socio-demographic characteristics, education and working hours, survivors of various diagnostic groups have lower incomes than siblings. Further research needs to identify the underlying causes.

## Introduction

Today, most childhood cancer patients (>80%) survive after treatment in developed countries[1, 2]. Late effects such as poor physical or mental health, functional impairments or activity limitations are well studied[3, 4]. Other factors are understudied, including the impact of childhood cancer on later earning capacity and income level. Income and educational attainment are linked, and studies have shown that cancer treatment during school years, combined with late effects, can lower the chance that survivors will excel at school[5–10]. But little is known about the later income situation of survivors. Income is a relevant factor for subjective well-being[11]. Some studies have assessed the personal income of survivors, but these included only small populations of survivors (N = 48–219)[12–16], or focused on specific diagnostic groups (osteosarcoma[17], retinoblastoma[18] or central nervous system [CNS] tumors and hematological malignancies[19]). Their results are inconsistent. Some studies found that income in survivors does not differ from controls[12, 15–17], and others found that their income is lower[14, 18, 19]. Researchers from the US Childhood Cancer Survivor Study investigated income and occupational outcomes in 4845 survivors of all diagnostic groups and 1727 siblings[20]. They found that survivors had earned less than siblings in all occupational fields. However, it remains unknown how underlying socio-demographic and clinical factors affect income in survivors.

Our goal was 1) to compare income between survivors and their siblings; 2) to assess the effects of socio-demographic characteristics on income in survivors and siblings (gender; age at survey; migration background; language region of Switzerland; parental education level; number of own children; work situation; personal education); 3) to compare income between survivors from different diagnostic groups, and to assess clinical characteristics on income in survivors (diagnosis, treatment modalities, age at diagnosis, relapse status).

## Methods

### The Swiss Childhood Cancer Survivor Study (SCCSS)

The Swiss Childhood Cancer Survivor Study (SCCSS) is a population-based, long-term follow-up study of all patients registered in the Swiss Childhood Cancer Registry (SCCR), who were diagnosed 1976–2005 at ≤21 years, and who survived ≥5 years after diagnosis.[21] The SCCR includes all children and adolescents in Switzerland diagnosed with leukemia, lymphoma, CNS tumors, malignant solid tumors, or Langerhans cell histiocytosis (LCH).[22] For this analysis, we included all survivors and siblings, who were aged ≥18 years at survey.

During 2007–2013, we traced addresses and sent a questionnaire to all survivors.[21, 23] Non-responders were mailed a second copy of the questionnaire. If they again failed to respond, we contacted them by phone. We asked survivors who were contacted in 2012 and earlier for their consent to contact their siblings for our comparison group. If survivors agreed, we sent the same questionnaire to siblings, without including cancer-related questions. Those who did not respond were sent another copy 4–6 weeks later, but were not contacted by phone. We used questionnaires similar to those used in US and UK childhood cancer survivor studies.[24, 25] We added questions about health behaviors and socio-demographic measures similar to those used in the Swiss Health Survey 2007[26] and the Swiss Census 2000.[27] Ethics approval was granted through the ethics committee of the canton of Bern to the SCCR.

### Assessment of income

We asked survivors and siblings to select one of the following categories to report their personal monthly net income: *≤ 3'000 Swiss Francs (CHF*); *3'001–4'500 CHF; 4'501–6'000 CHF*; *6'001–9'000 CHF*; and, *>9'000 CHF*. Net income was gross income from which social insurance and retirement insurance were subtracted. Net income includes income allocated for taxes and health insurance.

### Assessment of socio-demographic characteristics

Socio-demographic characteristics were divided into *baseline socio-demographic characteristics* (characteristics present already at birth), and *secondary socio-demographic characteristics* (characteristics that occurred after the cancer diagnosis) that may be influenced by cancer or its treatment.

The questionnaire assessed the following *baseline socio-demographic characteristics* for survivors and siblings: gender; age at survey; migration background; language region of Switzerland; and, parental education level. We considered participants who fulfilled one of the following criteria to have a migration background: not born in Switzerland; no Swiss citizenship at birth; or, at least one parent is not a Swiss citizen. Switzerland has different language regions, and health behaviors and mortality differ between them[28]. We coded the language region of participants as German, French, or Italian speaking. Personal and parental education level fell into three categories: compulsory schooling (≤9 years of schooling); secondary education (vocational training or upper secondary education); and, tertiary education (university or technical college education). We assessed the following *secondary socio-demographic characteristics* for survivors and siblings: number of own children; work situation; and, personal education. Participants were asked if they were employed and how many hours they worked per week. Unemployed participants were divided into these categories: educational training; receiving disability insurance (not on educational training); currently looking for a job (no educational training, no disability insurance); and, not looking for a job (no educational training, no disability insurance).

### Assessment of clinical data

The SCCR routinely collects clinical data. We extracted diagnosis, treatment modalities (surgery, chemotherapy, radiotherapy including the area of radiation, and bone marrow transplantation), age at diagnosis, and relapse status (yes/no) of survivors from the SCCR. We coded diagnoses according to the International Classification of Childhood Cancer, 3^rd^ edition (ICCC-3),[29] and put treatment modalities into hierarchical order for analysis: *chemotherapy* (may include surgery); *surgery only* (includes no other treatments), *bone marrow transplantation* (may include surgery and/or chemotherapy); and, *radiotherapy*, *not cranial* and *radiotherapy*, *including cranial* (may include surgery and/or chemotherapy and/or bone marrow transplantation).

The questionnaire asked survivors if their sight was severely impaired or if they were blind in one or both eyes, were deaf in one or both ears and if they had had a limb amputated. It also asked survivors if they currently experienced any kind of late effects of childhood cancer or its treatment (yes/no). We defined late effects as a physical or mental problem that resulted from the cancer or its treatment.

### Statistical analysis

First, we used Chi^2^ tests to compare *baseline* and *secondary socio-demographic characteristics* and income in survivors and siblings.

Second, we analyzed the association between socio-demographic characteristics and having a higher income (>4’500 CHF per month) with logistic regressions including survivors and siblings. We ran the analysis separately for *baseline socio-demographic characteristics* unaffected by cancer or its treatment (age, gender, language region, migration background and parental education), and for *secondary socio-demographic characteristics* that may be affected by the cancer or its treatment (having own children, weekly working hours and own education). We chose the income of >4’500 CHF as cut-off because it surpasses the recently discussed minimum wage of 4’000 CHF per month. We determined the effect of *baseline socio-demographic characteristics* on income by using univariable regressions. We included factors associated (p<0.05) with income in a multivariable logistic model. We also used univariable regressions to explore the association between *secondary socio-demographic characteristics* and income. We then included relevant (p<0.005) *baseline* and *secondary socio-demographic characteristics* associated with income in a multivariable logistic regression. Interaction of study group and gender was tested using likelihood ratio tests. We chose to test interaction for study group to find out if and how socio-demographic characteristics have a different effect on survivors than on siblings. We tested interaction for gender since it is known that income differences partially can be explained by gender.

Third, to find out if income differs between survivors of different types of tumors, we analyzed the association of diagnostic group with income in a multivariable logistic regression that included survivors and siblings. Siblings served as a reference group.

Fourth, we analyzed the association of clinical characteristics, including treatment, age at diagnosis, relapse status, blindness, deafness, amputation and perceived late effects, with income in a multivariable logistic regression that included only survivors.

We used the propensity score method[30] to standardize siblings to the survivor population for gender, age at survey, migration background and language region in all analyses that included siblings. We included robust variance estimation for clustered data to account for dependence of observations between survivors and their siblings. The criterion for statistical significance was a 2-sided p-value < 0.05. We used Stata, version 12.1 (StataCorp LP, College Station, TX) for all analyses.

## Results

### Socio-demographic characteristics

Of 4’111 survivors eligible for the SCCSS, we excluded 1’625 survivors for this study because they were aged <18 years at time of survey. Of the remaining 2’486 survivors address was not available for 129. Thus 2’357 survivors received our questionnaire. We used data from 1’506 survivors (response = 64%; S1 Table) and 598 siblings (response = 62%). Of the survivors, 48% were female, mean age at survey was 29.3 years (range: 18–55; SD = 7.5), and 26% had a migration background. Most survivors came from German speaking regions of Switzerland (70%, Table 1). The parents of most survivors had a secondary education (73%). We standardized the sibling population on age, gender, migration background, language region and parental education in accordance with the survivor population. More siblings (23%) than survivors (15%) had children (p<0.001). Fewer survivors (13%) reached tertiary education than siblings (21%; p<0.001). More siblings (84%) than survivors (78%) were employed (p = 0.016), with 56% of siblings and 57% of survivors in full time employment. Fewer siblings (10%) than survivors (13%) were in educational training.

**Table 1:** Socio-demographic characteristics of survivors and siblings.

	Survivors				Siblings				Siblings weighted^a^	
	N = 1’506				N = 598				N = 598	
	n		(%^b^)		n	(%^b^)		p^c^	(%^b^)	p^d^
**Baseline socio-demographic characteristics**										
**Female gender**	719		(48)		359	(60)		<0.001	(49)	0.693
**Age at survey (years)**								<0.001		
18-<25	490		(33)		116	(19)			(30)	0.727
25-<30	394		(26)		146	(24)			(28)	
30-<35	275		(18)		108	(18)			(18)	
35-<40	181		(12)		74	(12)			(12)	
≥40	166		(11)		154	(26)			(13)	
**Migration background**								<0.001		0.313
No	1117		(74)		506	(85)			(77)	
Yes	389		(26)		92	(15)			(23)	
**Language region**								<0.001		0.516
German	1054		(70)		483	(81)			(73)	
French	403		(27)		104	(17)			(25)	
Italian	49		(3)		11	(2)			(2)	
**Highest parental education**								0.541		0.882
Compulsory schooling	153		(11)		61	(11)			(10)	
Secondary education	1028		(73)		421	(75)			(74)	
Tertiary education	234		(17)		82	(15)			(16)	
**Secondary socio-demographic factors**										
**Do you have children?**								<0.001		<0.001
No		1’271		(84)	413		(69)		(77)	
Yes, 1–2 children		202		(13)	134		(22)		(18)	
Yes, 3 or more children		33		(2)	51		(9)		(5)	
**Personal education**								<0.001		<0.001
Compulsory schooling		127		(9)	0		(0)		(0)	
Secondary education		1150		(78)	460		(81)		(79)	
Tertiary education		194		(13)	107		(19)		(21)	
**Employment situation**								<0.001^e^		0.016
Employed for		1’174		(78)	516		(86)		(84)	
*≥40 hours per week (full time)*		*834*		*(57)*	*314*		*(53)*		*(56)*	
*30–39 hours per week*		*113*		*(8)*	*65*		*(11)*		*(8)*	
*20–29 hours per week*		*87*		*(6)*	*46*		*(8)*		*(7)*	
*10–19 hours per week*		*52*		*(4)*	*48*		*(8)*		*(8)*	
*0–9 hours per week*		*53*		*(4)*	*39*		*(7)*		*(6)*	
Not employed		332		(22)	82		(14)		(16)	
*Educational training*		*187*		*(13)*	*46*		*(8)*		*(10)*	
*Looking for a job*^f^		*52*		*(4)*	*20*		*(3)*		*(4)*	
*Not looking for a job*^f^		*35*		*(2)*	*12*		*(2)*		*(1)*	
*Disability insurance*^g^		*37*		*(2)*	*2*		*(<1)*		*(<1)*	
**Monthly income**										
**Income in CHF**								<0.001		0.002
≤ 3'000		580		(41)	205		(35)		(36)	
3'001–4’500		396		(28)	128		(22)		(24)	
4'501–6’000		271		(19)	149		(26)		(25)	
6'001–9’000		131		(9)	87		(15)		(13)	
>9’000		41		(3)	12		(2)		(2)	

NOTE: Percentages are based upon available data for each variable. Abbreviations: n, number; n.a., not applicable; CHF, Swiss Francs.

^a^Sibling population is standardized on age, gender, migration background, language region, parental education according to the survivor population. All numbers in siblings are based upon weighted percentages, therefore p-values do not apply to variables for which siblings were weighted

^b^Column percentages are given

^c^p-value calculated from Chi^2^ statistics that compare survivors and siblings

^d^p-value calculated from Chi^2^ statistics that compare survivors and weighted siblings

^e^p-value refers to comparison of survivors and siblings working vs. not working

^f^does not include those currently on educational training

^g^does not include those on educational training or working

The most frequent cancer diagnoses were leukemia (31%), lymphoma (22%) and CNS tumors (13%; Table 2).

**Table 2:** Clinical characteristics of survivors.

	Survivors N = 1’506	
	n	(%)^a^
**Diagnosis (ICCC-3)**		
I Leukemia	466	(31)
II Lymphoma	338	(22)
III CNS	197	(13)
IV Neuroblastoma	49	(3)
V Retinoblastoma	27	(2)
VI Renal tumor	67	(4)
VII Hepatic tumor	8	(1)
VIII Bone tumor	78	(5)
IX Soft tissue sarcoma	94	(6)
X Germ cell tumor	86	(6)
XI & XII Other tumors^b^	50	(3)
Langerhans cell histiocytosis	46	(3)
**Treatment**^c^		
Chemotherapy	1’149	(76)
Surgery	960	(64)
Radiotherapy		
No	924	(61)
Yes, excluding cranial	351	(23)
Yes, including cranial	231	(15)
Bone marrow transplantation	67	(4)
**Age at diagnosis (years)**		
0–5	471	(31)
>5–10	333	(22)
>10–15	488	(32)
>15–20	214	(14)
**Had relapse**	152	(11)
**Severe impairment or blindness on one or both eyes**	104	(7)
**Deaf on one or both ears**	10	(1)
**Amputation**	85	(6)
**Reported severe late effects from cancer**	548	(38)

NOTE: Percentages are based upon available data for each variable. Abbreviations: CNS, Central Nervous System; ICCC-3, International Classification of Childhood Cancer—Third Edition; n, number; n.a.

^a^Column percentages are given

^b^Other malignant epithelial neoplasms, malignant melanomas, and other or unspecified malignant neoplasms

^c^*“chemotherapy”* may include surgery, *“surgery only”* includes no other treatments, *“bone marrow transplantation*” may include surgery and/or chemotherapy, *“Radiotherapy*, *not cranial”* and *“Radiotherapy*, *including cranial”* may include surgery and/or chemotherapy and/or bone marrow transplantation.

### Income in survivors and siblings

Survivors had lower income than siblings (p = 0.002): More survivors than siblings reported a low monthly income of *≤ 3'000 CHF* (41% of survivors vs. 36% of siblings) and *3'001–4'500 CHF* (28% of survivors vs. 24% of siblings; Table 1). Fewer survivors than siblings reported a medium monthly income of *4'501–6'000 CHF* (19% of survivors vs. 25% of siblings) and *6'001–9'000 CHF* (9% of survivors vs. 13% of siblings). The proportion of survivors and siblings who reported a high income of *>9'000 CHF* was similar (3% of survivors vs. 2% of siblings).

### Socio-demographic characteristics associated with income

Results from logistic regressions showed that survivors were less likely to have a monthly income of >4’500 CHF than siblings, whether the analysis was unadjusted (OR = 0.54, p<0.001; Table 3, column A), adjusted for *baseline socio-demographic characteristics* (OR = 0.57, p = 0.001; Table 3, column B), or adjusted for *baseline* and *secondary socio-demographic characteristics* (OR = 0.46, p<0.001, Table 3, column C). Older participants (OR ranging from 4.23–11.90, p<0.001) and those with a tertiary education (OR = 2.14, p = 0.002) were more likely to have a monthly income of >4’500 CHF. Females (OR = 0.46, p<0.001), and those who worked less than 40 hours per week (OR ranging from 0.01–0.33, p<0.001) were less likely to have a monthly income of >4’500 CHF.

**Table 3:** Socio-demographic predictors of monthly income >4’500 CHF in survivors and siblings^a^.

	A) Univariable analysis			B) Multivariable analysis including baseline socio-demographic characteristics^b^			C)Multivariable analysis including baseline and secondary socio-demographic characteristics^c^		
	OR^d^	95% CI	p-value	OR^d^	95% CI	p-value	OR^d^	95% CI	p-value
***Study group***									
Sibling	1			1			1		
Survivor	0.54	0.43–0.69	<0.001	0.57	0.43–0.75	0.001	0.46	0.33–0.64	<0.001
**Basline socio-demographic characteristics (before cancer)**									
***Age at survey***									
18-<25 years	1			1		<0.001^e^	1		<0.001^e^
25-<30 years	5.65	3.49–9.15	<0.001	5.83	3.44–9.88	<0.001	4.31	2.52–7.39	<0.001
30-<35 years	9.22	5.62–15.15	<0.001	9.70	5.66–16.63	<0.001	7.05	4.08–12.19	<0.001
35-<40 years	10.87	6.38–18.53	<0.001	11.49	6.57–20.07	<0.001	10.23	5.68–18.41	<0.001
≥40 years	12.88	7.74–21.44	<0.001	11.86	6.78–20.73	<0.001	11.90	5.96–23.76	<0.001
***Gender***									
Male	1			1			1		
Female	0.31	0.24–0.40	<0.001	0.29	0.21–0.39	<0.001	0.46	0.32–0.65	<0.001
***Language region***									
German	1			n.a.			n.a.		
French/Italian	1.13	0.79–162	0.497						
***Migration***									
No	1			n.a.			n.a.		
Yes	0.85	0.59–1.21	0.361						
***Parental education***									
Compulsory schooling	0.73	0.43–1.25	0.251	0.51	0.27–0.96	0.038	0.57	0.32–1.02	0.057
Secondary education	1			1		0.043 ^e^	1		0.153^e^
Tertiary or university education	0.65	0.45–0.93	0.019	0.73	0.50–1.08	0.119	0.88	0.55–1.40	0.579
**Secondary socio-demographic characteristics (after cancer)**									
***Having children***									
No children	1			n.a.			1		0.364^e^
1 to 2 children	1.71	1.18–2.46	0.004				1.25	0.74–2.11	0.411
>2 children	1.22	0.63–2.35	0.562				0.71	0.36–1.40	0.323
***Working hours***									
≥40 hours	1			n.a.			1		<0.001^e^
30–39 hours	0.38	0.24–0.60	<0.001				0.33	0.19–0.59	<0.001
20–29 hours	0.07	0.04–0.15	<0.001				0.05	0.02–0.11	<0.001
10–19 hours	0.01	<0.01–0.07	<0.001				0.01	<0.01–0.06	<0.001
0–9 hours	0.04	0.02–0.07	<0.001				0.04	0.02–0.09	<0.001
***Personal education***									
Compulsory schooling	0.25	0.13–0.49	<0.001	n.a.			0.55	0.22–1.36	0.196
Secondary education	1						1		<0.002^e^
Tertiary or university education	3.01	2.07–4.39	<0.001				2.14	1.34–3.43	0.002

^a^Sibling population is standardized on age, gender, migration background and language region according to the survivor population

^b^Multivariable analysis, including baseline socio-demographic variables that were significant in the univariable analysis

^c^Multivariable analysis, including baseline socio-demographic variables and secondary socio-demographic characteristics that occurred after cancer was diagnosed and were significant in the univariable analysis

^d^OR for having a monthly income of >4500 CHF

^e^global p-value calculated with Wald test

**Fig 1:**
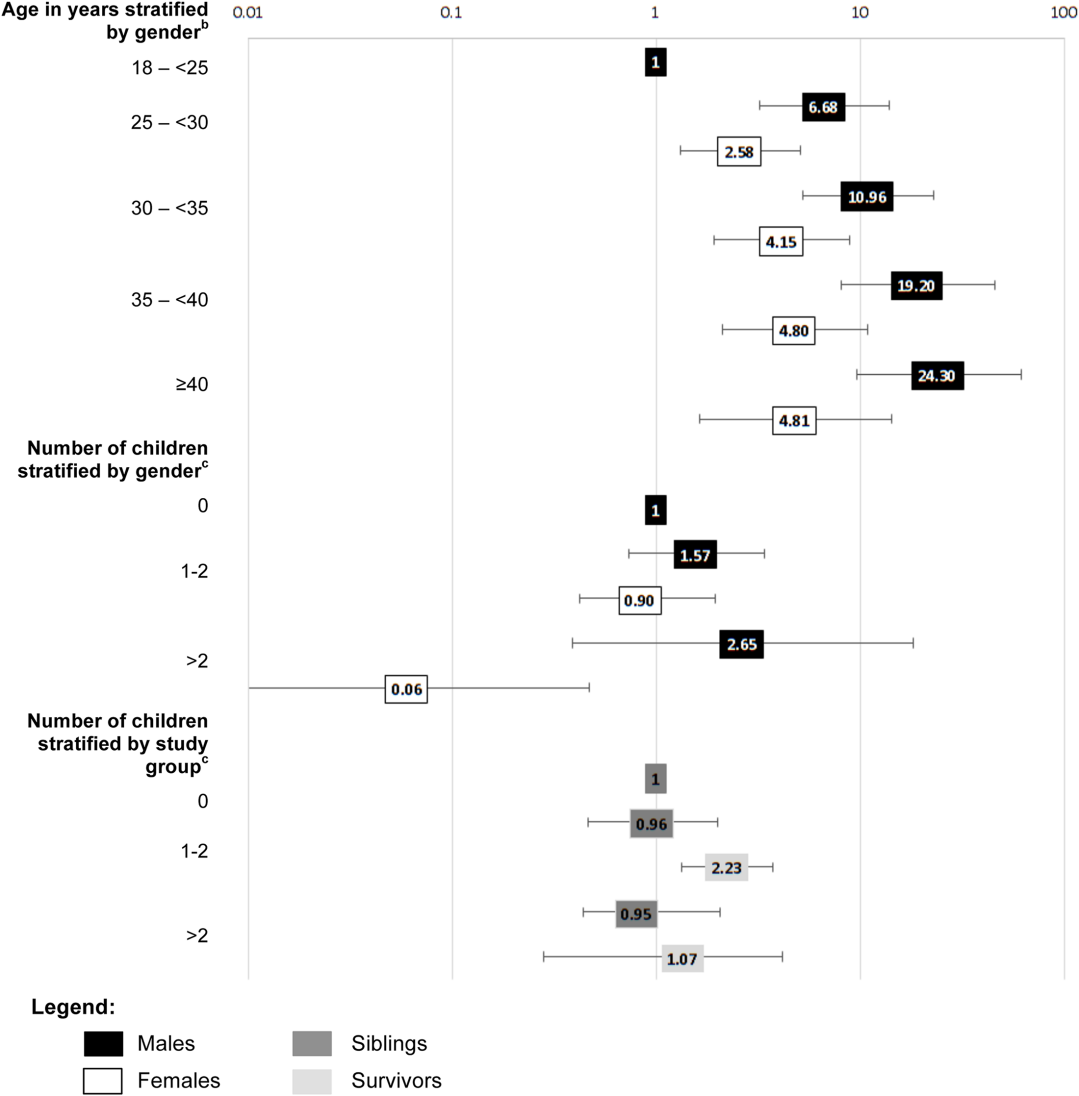
Interaction effects on socio-demographic predictors of monthly income >4’500 CHF. Fig 1 shows interaction effects of gender and study group on socio-demographic predictors of monthly income >4’500 CHF. ^a^Results were retrieved from multivariable logistic regression adjusted for baseline and secondary socio-demographic variables that were significant in the univariable model (Table 3). An OR<1 means that the respective group is less likely to have a monthly income of >4’500 CHF; ^b^Reference group are those aged 18 –<25 years; ^c^Reference group are those with 0 children.

Some predictors differed between females and males (S2 Table) and between survivors and siblings (S3 Table). Significant differences are displayed in Fig 1: Men’s income increased more steeply with age than women’s (p = 0.005). Women with >2 children were less likely to have an income of >4’500 CHF (OR = 0.06, p = 0.007) than men with no children. We also found an interaction between study groups and having own children (p = 0.035). Though survivors with 1–2 children were more likely than siblings with no children to have an income of >4’500 CHF (OR = 2.23, p = 0.002), income in siblings was not influenced by the number of children.

### Clinical characteristics associated with income

**Fig 2:**
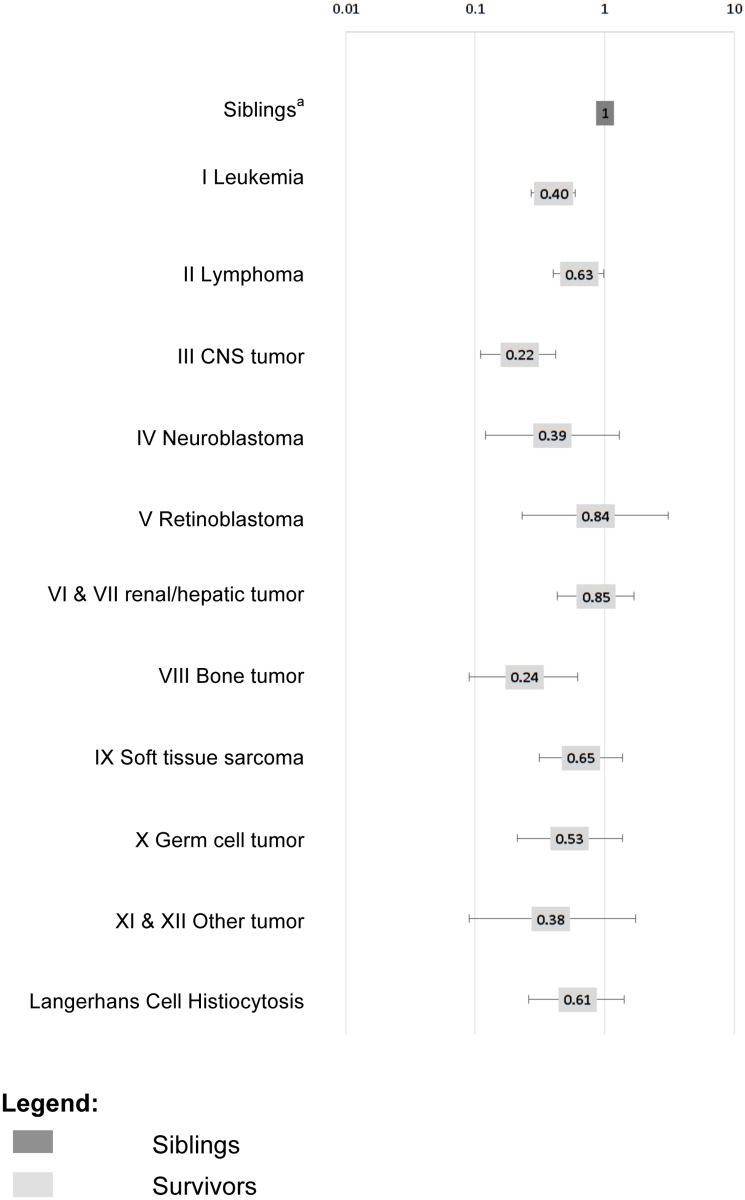
Association of diagnostic group with having a monthly income of >4’500 CHF. Fig 2 shows the association of diagnostic group with having a monthly income of >4’500 CHF compared to siblings. ^a^Multivariable analysis showing ORs adjusted for baseline and secondary socio-demographic variables that were significant in the univariable model (Table 3); ^b^Sibling population is standardized on age, gender, migration background and language region according to the survivor population.

After we adjusted for *baseline* and *secondary socio-demographic characteristics*, we found survivors of leukemia (OR = 0.40, p<0.001), lymphoma (OR = 0.63, p = 0.040), CNS tumors (OR = 0.22, p<0.001), and bone tumors (OR = 0.24, p = 0.003) were less likely than siblings to have a monthly income of >4’500 CHF (Fig 2).

Survivors treated by cranial irradiation (OR = 0.48, p = 0.006) were less likely than survivors treated by chemotherapy alone to have a monthly income of >4’500 CHF (Table 4). Those diagnosed aged >15–20 years were more likely than those diagnosed aged 0–5 years to have a monthly income of >4’500 CHF. Only in the univariable model were survivors who relapsed or had late effects less likely to have a higher income. All survivors who were deaf on one or both ears were in the lower income category.

**Table 4:** Association of clinical characteristics with having a monthly income of >4’500 CHF in survivors–results from univariable and multivariable logistic regression.

	Univariable analysis			Multivariable analysis^a^		
	OR	95%-CI	p-value	OR	95%-CI	p-value
***Treatment*** ^b^						
Chemotherapy	1			1		0.021^e^
Surgery only	1.13	0.79–1.61	0.514	1.42	0.72–2.77	0.306
Bone marrow transplant	0.72	0.26–1.98	0.525	1.56	0.34–7.06	0.567
Radiotherapy, not cranial	1.60	1.20–2.11	0.001	1.13	0.70–1.83	0.611
Radiotherapy, including cranial	0.74	0.51–1.07	0.107	0.48	0.29–0.81	0.006
***Age at diagnosis***						
0–5 years	1			1		0.073^e^
>5–10 years	1.41	1.02–1.96	0.038	1.61	0.98–2.63	0.060
>10–15 years	1.42	1.05–1.91	0.021	1.19	0.73–1.92	0.491
>15–20 years	3.67	2.58–5.23	<0.001	2.09	1.05–4.16	0.035
***Relapse***						
No	1			1		
Yes	0.65	0.43–0.98	0.038	0.69	0.38–1.25	0.222
***Blind on one or both eyes***						
No	1			n.a^c^		
Yes	0.74	0.48–1.14	0.173			
***Deaf on one or both ears***						
No	1			n.a^d^		
Yes	All have lower income.					
***Amputation***						
No	1			n.a^c^		
Yes	1.07	0.66–1.74	0.781			
***Perceived late effects***						
No	1			1		
Yes	0.77	0.61–0.98	0.034	0.92	0.63–1.34	0.665

^a^The analysis is adjusted for baseline and secondary socio-demographic variables that were significant in the univariable model (Table 3) and for diagnostic group

^b^*“chemotherapy”* may include surgery, *“surgery only”* includes no other treatments, *“bone marrow transplant*” may include surgery and/or chemotherapy, *“Radiotherapy*, *not cranial”* and *“Radiotherapy*, *including cranial”* may include surgery and/or chemotherapy and/or bone marrow transplantation

^c^Being blind in one or both eyes and amputation were not significantly associated (p-value was ≥0.05) with a monthly income of >4’500 CHF in the univariable model and were therefore not included in the multivariable model

^d^Being deaf on one or both ears perfectly predicts having an income of <4500 CHF, thus this variable could not be included into the model

^e^global p-value calculated with Wald test

## Discussion

This is the first study analyzing the association of socio-demographic and clinical characteristics with personal income in survivors. We found that income in survivors was lower than in siblings. Socio-demographic characteristics such as age, gender, working hours and education of the parents affect income. Survivors of leukemia, lymphoma, CNS tumors and bone tumors were likely to have an income lower than their siblings. Of survivors, those treated with cranial radiotherapy were most likely to have lower income.

### Income in survivors and comparison groups

Our results are in line with other studies. The US Childhood Cancer Survivor Study (CCSS) includes survivors diagnosed between 1970 and 1986. One study including 4’845 survivors and 1’727 siblings aged >25, all currently employed, found that survivors had a lower yearly income than siblings for all occupations[20]. The Norwegian Cancer Registry analyzed the yearly income of childhood cancer survivors of CNS tumors (n = 222) and hematological malignancies (n = 202), aged 25–44 years, and diagnosed between 1970–1997. More survivors of CNS tumors (14%) and hematological malignancies (15%) had a yearly income of <10’000 Euro than the general population (6%)[19]. Even after controlling for several socio-demographic factors affecting income (age, gender, parental education, personal education, working hours, number of own children) our study showed income to be lower in survivors than in siblings. Possible factors for low income in survivors compared to siblings might be that survivors make different career choices or receive different job offers than siblings.

### Socio-demographic characteristics associated with income

Few studies investigated how socio-demographic characteristics were associated with personal income. The CCSS[20] found that women were less likely in full-time managerial or professional occupations than males. Typically, full-time managerial or professional occupations are associated with high income compared to blue collar or service jobs. We also found that women and those with lower education have a lower income, which lines up with data from the general population of Switzerland, where men earn 24% more than women[31], and where higher education usually leads to higher income[32]. Thus the same factors associated with income observed in women from the general population also holds true for the population of survivors. Since our analysis controlled for various socio-demographic factors, lower incomes in survivors might be due to other factors such as personal career choice or discriminatory salary offers.

### Clinical characteristics associated with income

Few studies compared personal income between diagnostic groups. We found that survivors of CNS tumors had a lower income than siblings, in line with the results of the Norwegian study that compared survivors to the general population[19]. A US study found that survivors of CNS tumors earned less than other survivors and controls[13]. Cognitive late effects, caused by cranial irradiation or surgery might affect income in CNS survivors. We also found that survivors of leukemia, lymphoma and bone tumors also earned less than other survivors. For leukemia, this was also seen in Norway[19]. Further research needs to identify reasons for a lower income in leukemia survivors. We found no studies that explicitly examined income of lymphoma and bone tumor survivors, but it has been reported that lymphoma survivors can experience cognitive deficits from treatment[33]; and that amputee survivors of bone tumors can have deficits in education and employment[9].

Few studies assessed the effect of cranial irradiation on income. A Japanese study found that survivors treated with radiotherapy had lower annual incomes[12]. They believed the disparity was explained by a higher proportion of survivors who were studying at time of survey in the group of irradiated survivors. In the CCSS survivors treated with high dose cranial irradiation were less likely to be work in managerial or professional occupations than survivors treated by other means [20]. Survivors treated with cranial irradiation have more cognitive problems[34, 35] and thus lower educational achievements than healthy peers[36], all of which might contribute to lowering income.

We found that survivors diagnosed at age >15–20 years had higher incomes than survivors diagnosed at age 0–5 years. However, we did not find other studies that analyzed the effect of age at diagnosis on income. But others found lower occupational positions and education in survivors: The CCSS found that survivors diagnosed when they were younger were less often found in managerial or professional occupations than survivors diagnosed when they were older[20]. In a Swedish study, survivors of lymphoblastic leukemia with young age at diagnosis attained a lower level of education and were less often employed than controls[37]. Young age at diagnosis is a risk factor for cancer-related cognitive dysfunction[38]. Those cognitive deficits and being less often employed in managerial or professional occupations in survivors diagnosed at young age might lead to lower income.

### Clinical implication

Long treatment periods may explain why survivors earn less, since that pushes back their educational training and may cause them to start working later than their peers in the general population. Survivor income may also start to climb later than sibling income. In an earlier study, we found that the gap in educational achievements between survivors and the general population became smaller when we included those aged ≥27 years old[6]. Continuous research for a shorter and less toxic treatment of childhood cancer as well as educational support during and after treatment might help to improve and accelerate education, which might increase income. From our study we do not know if survivors choose jobs that pay less, if they are offered lower salaries than siblings when they apply for the same job or if specific late effects from treatment such as fatigue affects income. Further studies should include longitudinal assessment of income and career, and the employers’ attitude towards childhood cancer survivors.

### Limitations and Strengths

Because income was only assessed at one time point we could not measure changes of income levels over time. We could not estimate income in non-responders, so we could not determine if their income differed systematically from responders. However, almost all of our respondents answered our question about personal income (4% missing values in survivors and 2% in siblings). Our population-based approach was a strength. The distribution of diagnostic groups in our study was equivalent to the distribution in the Swiss population of childhood cancer survivors. However, non-responders differed from responders. Non-responders included more males, more survivors of lymphoma and CNS tumors, were less often treated with chemotherapy and older at diagnosis. We weighted the sibling population to maximize comparability to the survivor cohort. Stratifying analysis by diagnostic groups, and analyzing the effect of treatment and other cancer-related characteristics, allowed us to show specific survivor groups at risk for lower income.

### Conclusion

Survivors in a variety of diagnostic groups earn less than siblings, even after we adjusted for socio-demographic characteristics, education and working hours. Follow-up studies should investigate how income changes in survivors over time. Further they should assess underlying reasons for lower income beyond socio-demographic characteristics, including survivor’s preferences for certain job fields and how survivors are acting and being treated when their income is determined by employers.

## Supporting Information

S1 Table Non-responder responder analysis. (DOCX)

S2 Table Association of socio-demographic factors with having a monthly income of >4’500 CHF stratified by gender–results from multivariable logistic regression. (DOCX)

S3 Table Association of socio-demographic factors with having a monthly income of >4’500 CHF stratified by study group–results from multivariable logistic regression. (DOCX)
