# Supplementary material for: Income in Adult Survivors of Childhood Cancer
Source: PLoS One. 2016 May 23;11(5):e0155546. doi: 10.1371/journal.pone.0155546 (PMC4877063; doi:10.1371/journal.pone.0155546)
Supplement: S1 Table — (DOCX) [file pone.0155546.s001.docx]

**Table S1. Non-responder responder analysis**

|  | **Non-responders**  N=851 | | **Responders**  N=1’506 | |  |
| --- | --- | --- | --- | --- | --- |
|  | **n** | **(%)^a^** | **n** | **(%)^a^** | **p-value^d^** |
| **Female gender** | 349 | (41) | 719 | (48) | 0.002 |
| **Diagnosis (ICCC-3)** |  |  |  |  | <0.001 |
| I Leukemia | 166 | (20) | 466 | (31) |  |
| II Lymphoma | 238 | (28) | 338 | (22) |  |
| III CNS | 162 | (19) | 197 | (13) |  |
| IV Neuroblastoma | 17 | (2) | 49 | (3) |  |
| V Retinoblastoma | 12 | (1) | 27 | (2) |  |
| VI Renal tumor | 19 | (2) | 67 | (4) |  |
| VII Hepatic tumor | 7 | (1) | 8 | (1) |  |
| VIII Bone tumor | 58 | (7) | 78 | (5) |  |
| IX Soft tissue sarcoma | 72 | (8) | 94 | (6) |  |
| X Germ cell tumor | 36 | (4) | 86 | (6) |  |
| XI & XII Other tumors^b^ | 40 | (5) | 50 | (3) |  |
| Langerhans cell histiocytosis | 24 | (3) | 46 | (3) |  |
| **Treatment^c^** |  |  |  |  |  |
| Chemotherapy | 506 | (59) | 1’149 | (76) | <0.001 |
| Surgery | 573 | (67) | 960 | (64) | 0.079 |
| Radiotherapy |  |  |  |  | 0.071 |
| No | 554 | (65) | 924 | (61) |  |
| Yes, excluding cranial | 207 | (24) | 351 | (23) |  |
| Yes, including cranial | 90 | (11) | 231 | (15) |  |
| Bone marrow transplantation | 35 | (4) | 67 | (4) | 0.700 |
| **Age at diagnosis (years)** |  |  |  |  | <0.001 |
| 0-5 | 204 | (24) | 471 | (31) |  |
| >5-10 | 142 | (17) | 333 | (22) |  |
| >10-15 | 231 | (27) | 488 | (32) |  |
| >15-20 | 274 | (32) | 214 | (14) |  |
| **Had relapse** | 110 | (13) | 152 | (11) | 0.102 |

NOTE: Percentages are based upon available data for each variable. Abbreviations: CNS, Central Nervous System; ICCC-3, International Classification of Childhood Cancer - Third Edition; n, number; n.a. ^a^Column percentages are given; ^b^Other malignant epithelial neoplasms, malignant melanomas, and other or unspecified malignant neoplasms; ^c^*“chemotherapy”* may include surgery, *“surgery only”* includes no other treatments, *“bone marrow transplantation*” may include surgery and/or chemotherapy, *“Radiotherapy, not cranial”* and *“Radiotherapy, including cranial”* may include surgery and/or chemotherapy and/or bone marrow transplantation; ^d^p-value calculated from Chi^2^ statistics that compare non-responders and responders.
