# Supplementary material for: Income in Adult Survivors of Childhood Cancer
Source: PLoS One. 2016 May 23;11(5):e0155546. doi: 10.1371/journal.pone.0155546 (PMC4877063; doi:10.1371/journal.pone.0155546)
Supplement: S2 Table — (DOCX) [file pone.0155546.s002.docx]

**Table S2. Association of socio-demographic factors with having a monthly income of >4’500 CHF stratified by gender – results from multivariable logistic regression^a^**

|  |  | **Multivariable analysis**  **in *women* only** | | | **Multivariable analysis**  **in *men* only** | | | **Interaction gender** |
| --- | --- | --- | --- | --- | --- | --- | --- | --- |
|  |  | **OR^b^** | **95% CI** | **p-value** | **OR^b^** | **95% CI** | **p-value** | **p-value^d^** |
| ***Study group*** | Sibling | 1 |  |  | 1 |  |  | 0.143 |
|  | Survivor | 0.49 | 0.31 – 0.77 | 0.002 | 0.45 | 0.28 – 0.72 | 0.001 |  |
| **Baseline socio-demographic factors (before cancer)** | |  |  |  |  |  |  |  |
| ***Age at survey*** | 18-<25 years | 1 |  | *0.001^e^* | 1 |  | *<0.001^e^* | 0.005 |
|  | 25-<30 years | 2.58 | 1.31 – 5.09 | 0.004 | 6.68 | 3.20 – 13.94 | <0.001 |  |
|  | 30-<35 years | 4.15 | 1.93 – 8.91 | <0.001 | 10.96 | 5.25 – 22.89 | <0.001 |  |
|  | 35-<40 years | 4.80 | 2.11 – 10.90 | <0.001 | 19.20 | 8.07 – 45.67 | <0.001 |  |
|  | ≥40 years | 4.82 | 1.63 – 14.24 | <0.001 | 24.30 | 9.59 – 61.58 | <0.001 |  |
| ***Gender*** | Male | n.a.^c^ |  |  | n.a. ^c^ |  |  |  |
|  | Female |  |  |  |  |  |  |  |
| ***Language region*** | German | n.a.^c^ |  |  | n.a. ^c^ |  |  |  |
|  | French/Italian |  |  |  |  |  |  |  |
| ***Migration*** | No | n.a. ^c^ |  |  | n.a. ^c^ |  |  |  |
|  | Yes |  |  |  |  |  |  |  |
| ***Parental education*** | Compulsory schooling | 0.63 | 0.31 – 1.28 | 0.200 | 0.59 | 0.26 – 1.31 | 0.192 | 0.130 |
|  | Secondary education | 1 |  | *0.218^e^* | 1 |  | *0.372^e^* |  |
|  | Tertiary or university education | 0.60 | 0.29 – 1.27 | 0.180 | 1.13 | 0.60 – 2.12 | 0.709 |  |
| **Secondary socio-demographic factors (after cancer)** | |  |  |  |  |  |  |  |
| ***Having children*** | No children | 1 |  | *0.027^e^* | 1 |  | *0.332^e^* | <0.001 |
|  | 1 to 2 children | 0.90 | 0.42 – 1.95 | 0.798 | 1.57 | 0.73 – 3.40 | 0.251 |  |
|  | >2 children | 0.06 | 0.01 – 0.47 | 0.007 | 2.65 | 0.39 – 18.14 | 0.320 |  |
| ***Working hours*** | ≥40 hours | 1 |  | *<0.001^e^* | 1 |  | *<0.001^e^* | 0.836 |
|  | 30 - 39 hours | 0.37 | 0.21 – 0.66 | 0.001 | 0.40 | 0.12 – 1.31 | 0.130 |  |
|  | 20 - 29 hours | 0.07 | 0.02 – 0.18 | <0.001 | 0.06 | 0.02 – 0.25 | <0.001 |  |
|  | 10 - 19 hours | 0.02 | <0.01 – 0.12 | <0.001 | n.a. |  |  |  |
|  | 0 - 9 hours | 0.05 | 0.02 – 0.14 | <0.001 | 0.05 | 0.02 – 0.15 | <0.001 |  |
| ***Personal education*** | Compulsory schooling | 0.25 | 0.03 – 2.05 | 0.197 | 0.78 | 0.24 – 2.54 | 0.679 | 0.609 |
|  | Secondary education | 1 |  | *0.038^e^* | 1 |  | *0.036^e^* |  |
|  | Tertiary or university education | 2.01 | 1.06 – 3.84 | 0.033 | 2.40 | 1.21 – 4.78 | 0.012 |  |

^a^Sibling population is standardized on age, gender, migration background and language region according to the survivor population; ^b^OR for having a monthly income of >4500 CHF; ^c^Variable was not significantly associated (p-value was ≥0.05) with a monthly income of >4’500 CHF in the univariable full model (Table III) and therefore was not included in the multivariable model; ^d^p-value for interaction was calculated with likelihood ratio test.
