# Supplementary material for: Income in Adult Survivors of Childhood Cancer
Source: PLoS One. 2016 May 23;11(5):e0155546. doi: 10.1371/journal.pone.0155546 (PMC4877063; doi:10.1371/journal.pone.0155546)
Supplement: S3 Table — (DOCX) [file pone.0155546.s003.docx]

**Table S3. Association of socio-demographic factors with having a monthly income of >4’500 CHF stratified by study group – results from multivariable logistic regression^a^**

|  |  | **Multivariable analysis**  **in *survivors* only** | | | **Multivariable analysis**  **in *siblings* only** | | | **Interaction study group** |
| --- | --- | --- | --- | --- | --- | --- | --- | --- |
|  |  | **OR^b^** | **95% CI** | **p-value** | **OR^b^** | **95% CI** | **p-value** | **p-value^d^** |
| ***Study group*** | Sibling | n.a. |  |  | n.a. |  |  |  |
|  | Survivor |  |  |  |  |  |  |  |
| **Baseline socio-demographic factors (before cancer)** | |  |  |  |  |  |  |  |
| ***Age at survey*** | 18-<25 years | 1 |  | *<0.001^e^* | 1 |  | *<0.001^e^* | 0.318 |
|  | 25-<30 years | 4.43 | 2.76 – 7.12 | <0.001 | 4.25 | 1.82 – 9.93 | <0.001 |  |
|  | 30-<35 years | 7.63 | 4.57 – 12.74 | <0.001 | 7.32 | 2.92 – 18.37 | <0.001 |  |
|  | 35-<40 years | 12.82 | 7.18 – 22.89 | <0.001 | 8.22 | 3.11 – 21.71 | <0.001 |  |
|  | ≥40 years | 17.51 | 9.11 – 33.67 | <0.001 | 11.11 | 4.10 – 30.13 | <0.001 |  |
| ***Gender*** | Male | 1 |  |  | 1 |  |  | 0.121 |
|  | Female | 0.49 | 0.35 – 0.67 | <0.001 | 0.41 | 0.23 – 0.72 | 0.002 |  |
| ***Language region*** | German | n.a.^c^ |  |  | n.a. ^c^ |  |  |  |
|  | French/Italian |  |  |  |  |  |  |  |
| ***Migration*** | No | n.a. ^c^. |  |  | n.a. ^c^ |  |  |  |
|  | Yes |  |  |  |  |  |  |  |
| ***Parental education*** | Compulsory schooling | 0.67 | 0.43 – 1.06 | 0.085 | 0.55 | 0.21 – 1.44 | 0.224 | 0.307 |
|  | Secondary education | 1 |  | *0.200^e^* | 1 |  | *0.395^e^* |  |
|  | Tertiary or university education | 1.07 | 0.65 – 1.78 | 0.769 | 0.76 | 0.37 – 1.55 | 0.445 |  |
| **Secondary socio-demographic factors (after cancer)** | |  |  |  |  |  |  |  |
| ***Having children*** | No children | 1 |  | *0.009^e^* | 1 |  | *0.988^e^* | 0.035 |
|  | 1 to 2 children | 2.23 | 1.34 – 3.71 | 0.002 | 0.96 | 0.46 – 1.99 | 0.913 |  |
|  | >2 children | 1.07 | 0.28 – 4.14 | 0.917 | 0.95 | 0.44 – 2.05 | 0.888 |  |
| ***Working hours*** | ≥40 hours | 1 |  | *<0.001^e^* | 1 |  | *<0.001^e^* | 0.696 |
|  | 30 - 39 hours | 0.32 | 0.19 – 0.55 | <0.001 | 0.40 | 0.19 – 0.86 | <0.001 |  |
|  | 20 - 29 hours | 0.07 | 0.03 – 0.17 | <0.001 | 0.04 | 0.02 – 0.12 | <0.001 |  |
|  | 10 - 19 hours | 0.01 | <0.01 – 0.08 | <0.001 | 0.02 | <0.01 – 0.10 | <0.001 |  |
|  | 0 - 9 hours | 0.02 | 0.01 – 0.06 | <0.001 | 0.05 | 0.02 – 0.12 | <0.001 |  |
| ***Personal education*** | Compulsory schooling | 0.49 | 0.22 – 1.10 | 0.083 | n.a. |  |  | 0.287 |
|  | Secondary education | 1 |  | *<0.001^e^* | 1 |  | *n.a.^e^* |  |
|  | Tertiary or university education | 2.44 | 1.59 – 3.73 | <0.001 | 2.20 | 1.09 – 4.42 | 0.027 |  |

^a^Sibling population is standardized on age, gender, migration background and language region according to the survivor population; ^b^OR for having a monthly income of >4500 CHF; ^c^Variable was not significantly associated (p-value was ≥0.05) with a monthly income of >4’500 CHF in the univariable full model (Table III) and therefore was not included in the multivariable model; ^d^p-value for interaction was calculated with likelihood ratio test. ^e^global p-values.
